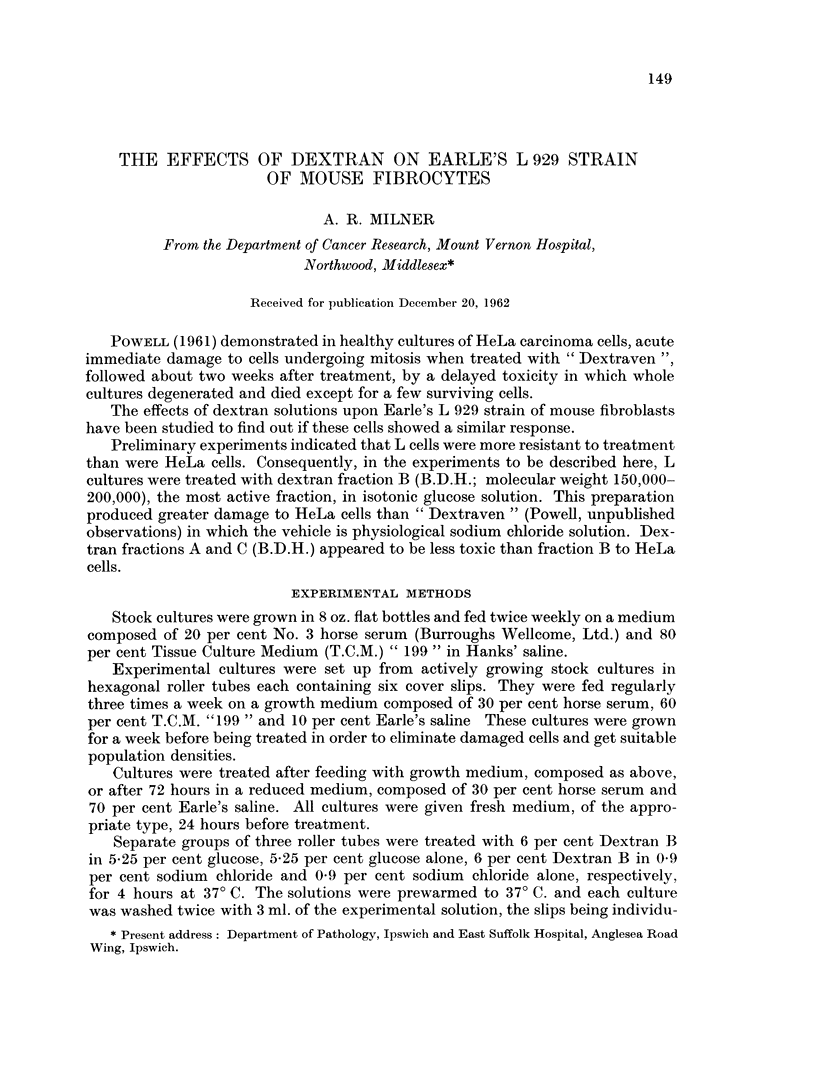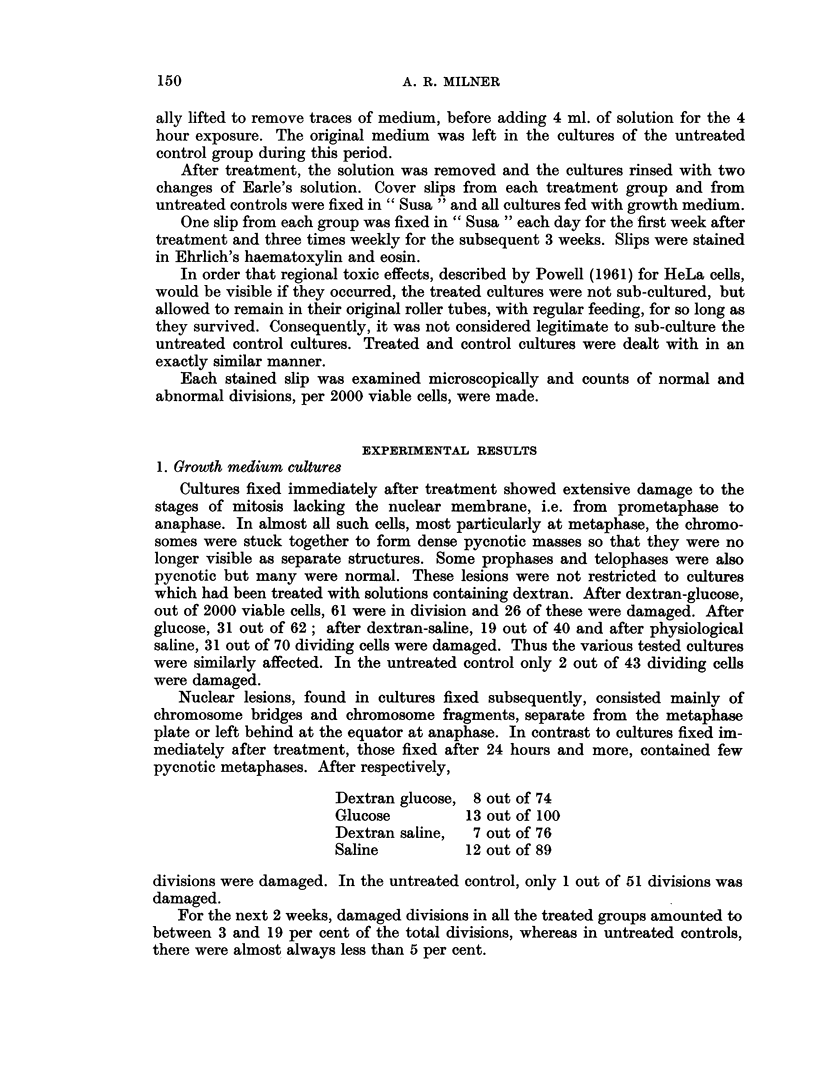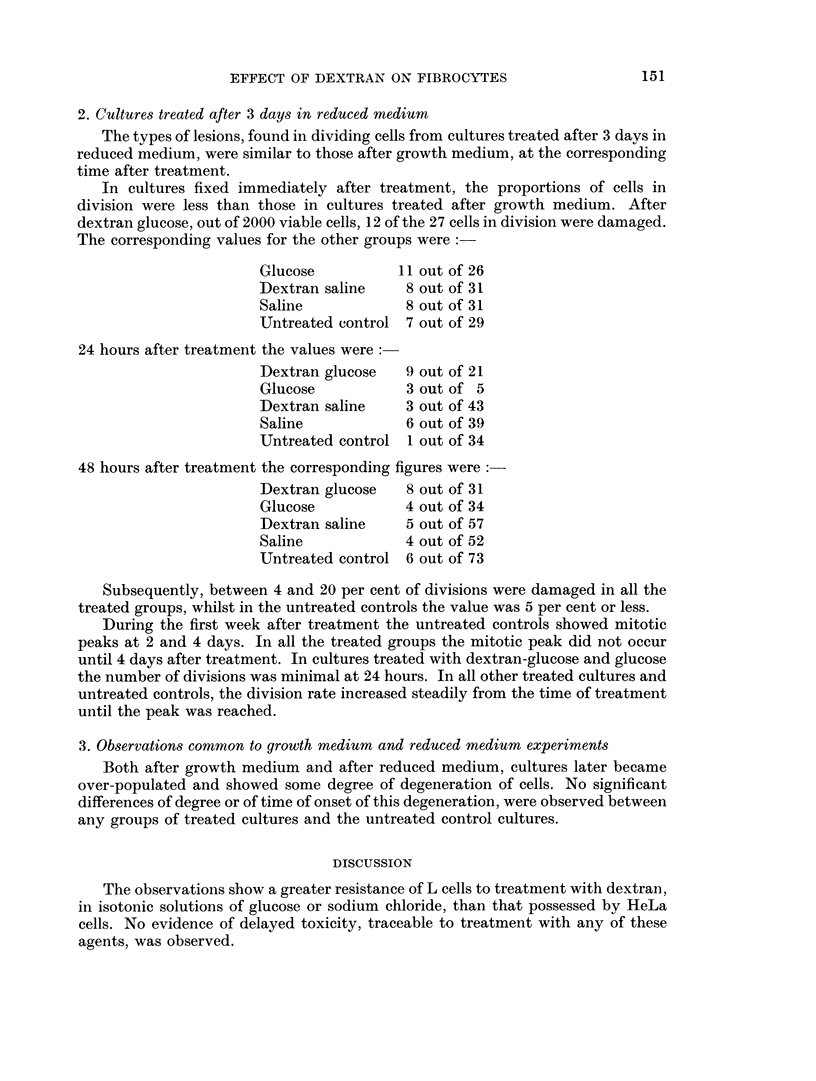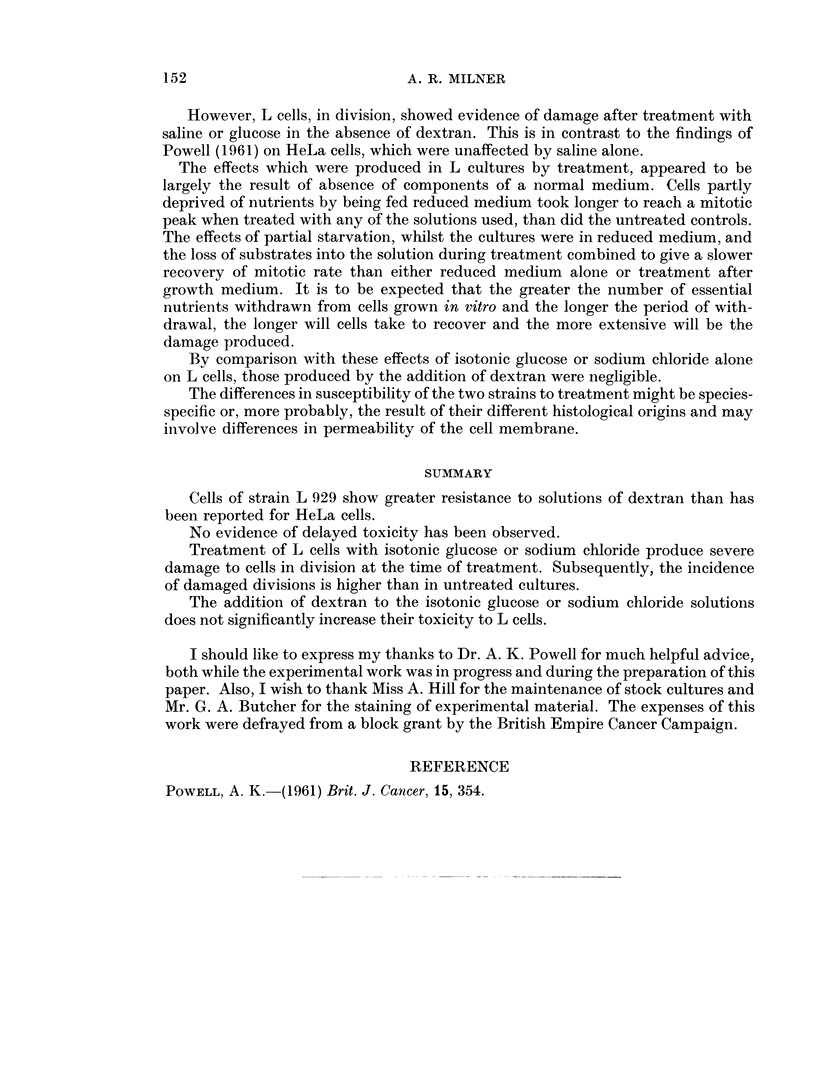# The Effects of Dextran on Earle's L 929 Strain of Mouse Fibrocytes

**DOI:** 10.1038/bjc.1963.22

**Published:** 1963-03

**Authors:** A. R. Milner


### THE EFFECTS OF DEXTRAN ON EARLE'S L 929 STRAIN OF MOUSE FIBROCYTES

A. R. MILNER

*From the Department of Cancer Research, Mount Vernon Hospital, Northwood, Middlesex**

Received for publication December 20, 1962

POWELL (1961) demonstrated in healthy cultures of HeLa carcinoma cells, acute immediate damage to cells undergoing mitosis when treated with " Dextraven ", followed about two weeks after treatment, by a delayed toxicity in which whole cultures degenerated and died except for a few surviving cells.

The effects of dextran solutions upon Earle's L 929 strain of mouse fibroblasts have been studied to find out if these cells showed a similar response.

Preliminary experiments indicated that L cells were more resistant to treatment than were HeLa cells. Consequently, in the experiments to be described here, L cultures were treated with dextran fraction B (B.D.H.; molecular weight 150,000–200,000), the most active fraction, in isotonic glucose solution. This preparation produced greater damage to HeLa cells than " Dextraven " (Powell, unpublished observations) in which the vehicle is physiological sodium chloride solution. Dextran fractions A and C (B.D.H.) appeared to be less toxic than fraction B to HeLa cells.

### EXPERIMENTAL METHODS

Stock cultures were grown in 8 oz. flat bottles and fed twice weekly on a medium composed of 20 per cent No. 3 horse serum (Burroughs Wellcome, Ltd.) and 80 per cent Tissue Culture Medium (T.C.M.) " 199 " in Hanks' saline.

Experimental cultures were set up from actively growing stock cultures in hexagonal roller tubes each containing six cover slips. They were fed regularly three times a week on a growth medium composed of 30 per cent horse serum, 60 per cent T.C.M. "199 " and 10 per cent Earle's saline These cultures were grown for a week before being treated in order to eliminate damaged cells and get suitable population densities.

Cultures were treated after feeding with growth medium, composed as above, or after 72 hours in a reduced medium, composed of 30 per cent horse serum and 70 per cent Earle's saline. All cultures were given fresh medium, of the appropriate type, 24 hours before treatment.

Separate groups of three roller tubes were treated with 6 per cent Dextran B in 5·25 per cent glucose, 5·25 per cent glucose alone, 6 per cent Dextran B in 0·9 per cent sodium chloride and 0·9 per cent sodium chloride alone, respectively, for 4 hours at 37° C. The solutions were prewarmed to 37° C. and each culture was washed twice with 3 ml. of the experimental solution, the slips being individu-

* Present address : Department of Pathology, Ipswich and East Suffolk Hospital, Anglesea Road Wing, Ipswich.

ally lifted to remove traces of medium, before adding 4 ml. of solution for the 4 hour exposure. The original medium was left in the cultures of the untreated control group during this period.

After treatment, the solution was removed and the cultures rinsed with two changes of Earle's solution. Cover slips from each treatment group and from untreated controls were fixed in " Susa " and all cultures fed with growth medium.

One slip from each group was fixed in " Susa " each day for the first week after treatment and three times weekly for the subsequent 3 weeks. Slips were stained in Ehrlich's haematoxylin and eosin.

In order that regional toxic effects, described by Powell (1961) for HeLa cells, would be visible if they occurred, the treated cultures were not sub-cultured, but allowed to remain in their original roller tubes, with regular feeding, for so long as they survived. Consequently, it was not considered legitimate to sub-culture the untreated control cultures. Treated and control cultures were dealt with in an exactly similar manner.

Each stained slip was examined microscopically and counts of normal and abnormal divisions, per 2000 viable cells, were made.

### EXPERIMENTAL RESULTS

### 1. *Growth medium cultures*

Cultures fixed immediately after treatment showed extensive damage to the stages of mitosis lacking the nuclear membrane, i.e. from prometaphase to anaphase. In almost all such cells, most particularly at metaphase, the chromosomes were stuck together to form dense pycnotic masses so that they were no longer visible as separate structures. Some prophases and telophases were also pycnotic but many were normal. These lesions were not restricted to cultures which had been treated with solutions containing dextran. After dextran-glucose, out of 2000 viable cells, 61 were in division and 26 of these were damaged. After glucose, 31 out of 62 ; after dextran-saline, 19 out of 40 and after physiological saline, 31 out of 70 dividing cells were damaged. Thus the various tested cultures were similarly affected. In the untreated control only 2 out of 43 dividing cells were damaged.

Nuclear lesions, found in cultures fixed subsequently, consisted mainly of chromosome bridges and chromosome fragments, separate from the metaphase plate or left behind at the equator at anaphase. In contrast to cultures fixed immediately after treatment, those fixed after 24 hours and more, contained few pycnotic metaphases. After respectively,

| | |
|---|---|
| Dextran glucose, | 8 out of 74 |
| Glucose | 13 out of 100 |
| Dextran saline, | 7 out of 76 |
| Saline | 12 out of 89 |

divisions were damaged. In the untreated control, only 1 out of 51 divisions was damaged.

For the next 2 weeks, damaged divisions in all the treated groups amounted to between 3 and 19 per cent of the total divisions, whereas in untreated controls, there were almost always less than 5 per cent.

2. *Cultures treated after 3 days in reduced medium*

The types of lesions, found in dividing cells from cultures treated after 3 days in reduced medium, were similar to those after growth medium, at the corresponding time after treatment.

In cultures fixed immediately after treatment, the proportions of cells in division were less than those in cultures treated after growth medium. After dextran glucose, out of 2000 viable cells, 12 of the 27 cells in division were damaged. The corresponding values for the other groups were :—

| | |
|---|---|
| Glucose | 11 out of 26 |
| Dextran saline | 8 out of 31 |
| Saline | 8 out of 31 |
| Untreated control | 7 out of 29 |

24 hours after treatment the values were :—

| | |
|---|---|
| Dextran glucose | 9 out of 21 |
| Glucose | 3 out of 5 |
| Dextran saline | 3 out of 43 |
| Saline | 6 out of 39 |
| Untreated control | 1 out of 34 |

48 hours after treatment the corresponding figures were :—

| | |
|---|---|
| Dextran glucose | 8 out of 31 |
| Glucose | 4 out of 34 |
| Dextran saline | 5 out of 57 |
| Saline | 4 out of 52 |
| Untreated control | 6 out of 73 |

Subsequently, between 4 and 20 per cent of divisions were damaged in all the treated groups, whilst in the untreated controls the value was 5 per cent or less.

During the first week after treatment the untreated controls showed mitotic peaks at 2 and 4 days. In all the treated groups the mitotic peak did not occur until 4 days after treatment. In cultures treated with dextran-glucose and glucose the number of divisions was minimal at 24 hours. In all other treated cultures and untreated controls, the division rate increased steadily from the time of treatment until the peak was reached.

3. *Observations common to growth medium and reduced medium experiments*

Both after growth medium and after reduced medium, cultures later became over-populated and showed some degree of degeneration of cells. No significant differences of degree or of time of onset of this degeneration, were observed between any groups of treated cultures and the untreated control cultures.

### DISCUSSION

The observations show a greater resistance of L cells to treatment with dextran, in isotonic solutions of glucose or sodium chloride, than that possessed by HeLa cells. No evidence of delayed toxicity, traceable to treatment with any of these agents, was observed.

However, L cells, in division, showed evidence of damage after treatment with saline or glucose in the absence of dextran. This is in contrast to the findings of Powell (1961) on HeLa cells, which were unaffected by saline alone.

The effects which were produced in L cultures by treatment, appeared to be largely the result of absence of components of a normal medium. Cells partly deprived of nutrients by being fed reduced medium took longer to reach a mitotic peak when treated with any of the solutions used, than did the untreated controls. The effects of partial starvation, whilst the cultures were in reduced medium, and the loss of substrates into the solution during treatment combined to give a slower recovery of mitotic rate than either reduced medium alone or treatment after growth medium. It is to be expected that the greater the number of essential nutrients withdrawn from cells grown *in vitro* and the longer the period of withdrawal, the longer will cells take to recover and the more extensive will be the damage produced.

By comparison with these effects of isotonic glucose or sodium chloride alone on L cells, those produced by the addition of dextran were negligible.

The differences in susceptibility of the two strains to treatment might be species-specific or, more probably, the result of their different histological origins and may involve differences in permeability of the cell membrane.

### SUMMARY

Cells of strain L 929 show greater resistance to solutions of dextran than has been reported for HeLa cells.

No evidence of delayed toxicity has been observed.

Treatment of L cells with isotonic glucose or sodium chloride produce severe damage to cells in division at the time of treatment. Subsequently, the incidence of damaged divisions is higher than in untreated cultures.

The addition of dextran to the isotonic glucose or sodium chloride solutions does not significantly increase their toxicity to L cells.

I should like to express my thanks to Dr. A. K. Powell for much helpful advice, both while the experimental work was in progress and during the preparation of this paper. Also, I wish to thank Miss A. Hill for the maintenance of stock cultures and Mr. G. A. Butcher for the staining of experimental material. The expenses of this work were defrayed from a block grant by the British Empire Cancer Campaign.

### REFERENCE

POWELL, A. K.—(1961) *Brit. J. Cancer*, **15**, 354.